# Optimizing diesel engine performance and emissions with mahua biodiesel blends using taguchi methodology

**DOI:** 10.1371/journal.pone.0332035

**Published:** 2025-09-05

**Authors:** G. Praveen Kumar Yadav, Din Bandhu, Suman Chatterjee, Prashant Kumar Gangwar

**Affiliations:** 1 Department of Mechanical Engineering, G Pulla Reddy Engineering College, Kurnool, Andhra Pradesh, India; 2 Department of Mechanical Engineering, Galgotias University, Greater Noida, Uttar Pradesh, India; 3 I-Form, Advanced Manufacturing Research Centre, Dublin City University, Dublin, Ireland; 4 Department of Construction Technology & Management, Woldia University, Woldia City, Ethiopia; Universiti Sains Malaysia, MALAYSIA

## Abstract

This study investigates how adjusting operational parameters influences the performance and emission characteristics of a diesel engine operating on a blend of traditional diesel fuel and mahua biodiesel. The biodiesel was obtained using the transesterification method, and fuel blends were formulated with diesel proportions ranging from 80% to 100% and biodiesel content from 0% to 20%. Key engine parameters such as engine load (20 –100%), mahua biodiesel blend (0 –20%), and engine speed (1300 –1450 rpm) were varied systematically during the experiments, while the compression ratio was held constant at 18:1. The aim was to determine the most effective combination of settings to enhance combustion efficiency and reduce harmful exhaust emissions. The findings demonstrated a modest yet meaningful improvement in engine efficiency, with gains of approximately 2–3%. While seemingly incremental, this enhancement becomes significant when coupled with concurrent reductions in harmful emissions, aligning with global efforts to transition toward sustainable fuel alternatives. The optimized biodiesel blends not only improved fuel utilization but also advanced environmental objectives by mitigating pollutant emissions, underscoring their dual role in enhancing performance and ecological sustainability. The outcomes of this study support the potential of mahua biodiesel as a viable and eco-friendly supplement to petroleum diesel. Overall, the results contribute meaningful data toward improving diesel engine performance while reducing environmental pollution.

## Introduction

Diesel fuel is typically derived from fossil fuels through a process that involves heating to temperatures around 300°C. Its widespread use in industries stems from its ability to deliver high torque and power, as well as its easy availability [1]. However, numerous studies in recent years have highlighted the environmental drawbacks of diesel engines. These engines emit pollutants such as CO, CO_2_, NOx, and HCs, all of which contribute to air pollution and climate change [2]. With concerns rising over the environmental impact and the finite nature of fossil fuel reserves, the need for alternative fuels has become increasingly urgent. Identifying a reliable replacement for diesel, supported by a sustainable and abundant source of raw materials, is crucial for reducing emissions while maintaining engine performance [3]. Several alternative fuels have been explored in the search for a cleaner substitute to conventional diesel, including vegetable oils, fish oils, and Karanja-based biodiesel. However, one of the key considerations in this pursuit is ensuring that the performance and efficiency of diesel engines remain uncompromised [4]. For any biodiesel to be a viable option, it must not only reduce harmful emissions but also sustain engine output.

Additionally, the chosen fuel must be readily available, as diesel is consumed in vast quantities across multiple sectors. Economic feasibility remains critical for adoption, especially in regions where smallholder farmers supply feedstocks [5]. In this context, non-edible biodiesel sources that are abundantly available offer a distinct advantage over others [6]. Mahua oil, in particular, emerges as a promising candidate due to its wide availability and non-edible nature. Non-edible sources like mahua align with circular bioeconomy principles by minimizing land-use conflicts and supporting rural livelihoods. The process of transesterification, where triglycerides react with alcohol, converts the oil into biodiesel, with glycerol as a by-product [7–9]. This method has been shown to improve the calorific value of the resulting fuel, enabling it to produce more energy and blend efficiently with conventional diesel. Moreover, the oxygen-rich composition of biodiesel contributes to more complete combustion in diesel engines, leading to a noticeable reduction in CO emissions [10]. Recent studies have highlighted the growing need to adapt existing engine systems for compatibility with sustainable fuels. For instance, research on marine diesel engine adjustments has outlined critical modifications necessary to accommodate alternative fuels, emphasizing operational reliability and emissions control [11]. Similarly, the integration of alcohols such as butanol into biodiesel blends has shown improvements in combustion quality and a measurable reduction in regulated emissions [12]. Further advances include the use of graphene quantum dot additives in waste-derived fuels, which have been shown to stabilize combustion and reduce harmful exhaust emissions in blended fuel applications [13]. These recent findings reinforce the importance of optimizing fuel formulations and engine parameters, particularly when incorporating oxygenated biofuels like mahua biodiesel. Our study builds on this growing body of work by providing a structured optimization of engine operating conditions using a Taguchi-based approach tailored specifically for mahua-based biodiesel blends. Recent studies underscore the need for robust optimization frameworks to balance combustion efficiency and emissions in biodiesel engines, particularly when using non-edible feedstocks [14].

For biodiesel to be a practical alternative, it must work with existing diesel engines without requiring major modifications. Key factors like injection timing, compression ratio, and indicated pressure significantly influence engine performance when using biodiesel blends [15]. Since engine efficiency is critical, a reliable method such as the Taguchi technique is often used to optimize operating conditions. Studies show this approach effectively improves performance and reduces emissions [16]. Biodiesel blends, made from renewable sources like vegetable oils and animal fats, offer a cleaner option while maintaining compatibility with current fuel systems. Ensuring smooth integration and stable engine operation is essential for reducing emissions and supporting a shift toward sustainable fuel use [17]. This study investigates how different biodiesel blend ratios and power levels influence diesel engine performance and emissions. The goal is to identify operating conditions that enhance efficiency while lowering harmful emissions, promoting both performance and environmental sustainability. A compression ratio of 18 was used, as higher compression is known to improve combustion quality [18]. Various blends ranging from pure diesel (D100) to 20% biodiesel (D80B20) were tested. The results helped determine the most effective blend that maintains engine efficiency and supports cleaner operation.

In a study conducted by Kishore, Dhana Raju, and Kolli [19], experiments were carried out to improve the performance of a diesel engine using a blend of 20% Tamarind Seed Methyl Ester (TSME) with 80% conventional diesel. The research focused on identifying the most effective combination of intake parameters, namely, injection pressure, injection timing, and exhaust gas recirculation (EGR), to enhance engine performance. Each parameter was tested at three distinct levels. Key performance indicators included brake thermal efficiency, brake-specific fuel consumption, hydrocarbon emissions, nitrogen oxide levels, and smoke opacity. To efficiently handle the experimental design and reduce the total number of trials, the researchers applied the Taguchi L_27_ orthogonal array method. This approach is known for its ability to analyze the impact of multiple variables simultaneously with fewer experiments. In addition, analysis of variance (ANOVA) was used to assess how much each parameter influenced the engine’s output. The results showed that injection timing played a major role in improving thermal efficiency. At the same time, EGR had the greatest effect on controlling nitrogen oxide emissions and smoke, with injection timing and pressure also contributing. Wilson [20] aimed to reduce nitrogen oxide emissions and improve fuel efficiency in a single-cylinder, direct-injection diesel engine through experimental testing and statistical optimization. Using a 5.2 kW engine, five parameters—clearance volume, valve opening pressure, nozzle diameter, injection timing, and load torque—were tested at four different levels. The Taguchi method was employed to analyze the influence of these parameters on NOx output and brake-specific fuel consumption. Based on signal-to-noise ratio analysis, the best combination of settings was identified. Final validation tests closely matched the predicted results, confirming the method’s effectiveness in linking engine settings to emission and efficiency outcomes.

This work explores the performance and emission behavior of a diesel engine fuelled with mahua biodiesel blends, focusing on practical operating conditions and real-world applicability. Mahua oil, a non-edible and regionally abundant feedstock, has not been extensively studied in this context, particularly using a systematic optimization approach. The study employs the Taguchi design method to evaluate the combined effects of engine load, fuel blend ratio, and speed, each varied across five levels. Unlike previous studies that often isolate performance or emissions, this research examines both simultaneously across six response parameters, offering a broader understanding of engine behavior. The use of commercially prepared biodiesel without further processing ensures the results are relevant to real-world scenarios. This comprehensive evaluation helps identify suitable operating conditions for integrating mahua biodiesel into existing diesel engines without major modifications, which is particularly useful for rural and decentralized energy systems. Moreover, this study contributes uniquely to the field by focusing on mahua oil, a non-edible, regionally abundant, and underexplored biodiesel feedstock with direct applicability in rural energy contexts. Unlike many studies that consider only single response parameters or purified fuels, this work uses commercially available biodiesel. It applies a structured Taguchi L_25_ orthogonal array to simultaneously optimize engine load, speed, and blend ratio across six critical performance and emission metrics.

Additionally, the study presents normalized emissions for practical comparability. This comprehensive approach fills a gap in existing literature by combining material novelty, methodological rigor, and real-world relevance in biodiesel-diesel engine optimization. This work aims to examine the impact of varying engine load, biodiesel blend percentage, and engine speed on the overall performance and emission behavior of a diesel engine fueled with mahua biodiesel–diesel mixtures.

### Fuel preparation

The biodiesel used in this study was sourced commercially and did not undergo additional purification. It was produced through the transesterification process, where triglycerides from vegetable oils or animal fats react with methanol in the presence of a catalyst like potassium hydroxide [21]. This reaction yields fatty acid methyl esters, known as biodiesel, and glycerol as a by-product. The process effectively separates fuel-grade esters from raw oil, resulting in a product with properties similar to conventional diesel. Biodiesel produced this way is biodegradable, has clean-burning characteristics, and emits fewer pollutants such as carbon monoxide, sulfur compounds, and particulate matter [22]. However, unmodified biodiesels may exhibit suboptimal cold-flow properties, potentially affecting fuel injection in colder climates [23]. Due to its compatibility with existing diesel engines and infrastructure, biodiesel derived from transesterification is a practical, renewable alternative that supports efforts to reduce greenhouse gas emissions in the transport sector.

### Experimental setup

**Fig 1:**
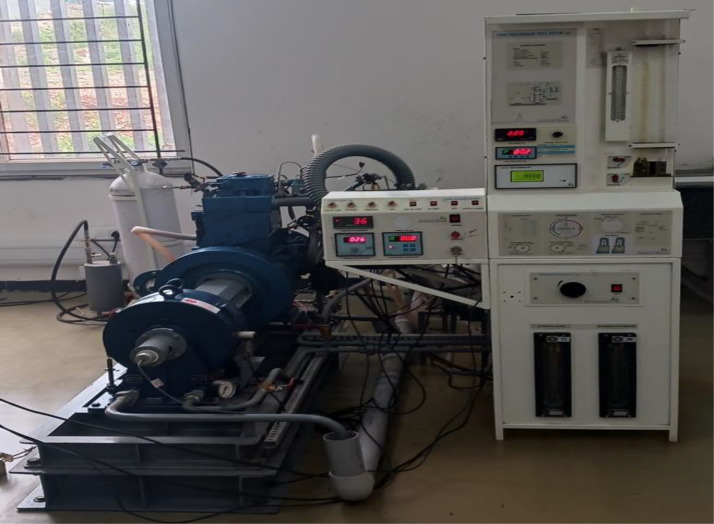
Computerized variable compression ratio diesel engine.

The experimental setup involved a single-cylinder, four-stroke, direct-injection diesel engine coupled with an eddy current dynamometer, which is shown in Table 1 and Fig 1. This type of dynamometer was chosen for its long lifespan and minimal maintenance needs, as it operates without friction-based components. It enabled controlled variation of engine load, allowing for performance evaluation under different power conditions [24]. Engine behavior and emissions were closely monitored using IC Engine Combustion Analysis software, which provided detailed data on efficiency and combustion at various loads and blend ratios. Additionally, a gas analyzer was used to measure emissions of CO, CO₂, NOx, and HC, offering insights into the environmental effects of fuel combustion [25]. This systematic approach helped assess how load conditions and biodiesel blends influence engine performance and emissions in a controlled laboratory setting, supporting efforts to improve efficiency and reduce environmental impact.

**Table 1:** Engine specifications.

Number of Cylinders	1
Number of Strokes	4
Stroke Length	110 mm
Connecting rod length	234 mm
Orifice Diameter	20 mm
Dynamometer length	185 mm
Fuel	Diesel
Power	3.5 kW
Compression Ratio	18
Dynamometer Type	Eddy Current

For each test condition, the engine was operated until it reached a steady state, typically after 5 minutes of continuous operation, to ensure consistency in measurement. Data for each parameter, such as torque, fuel consumption, and exhaust emissions, were recorded three times per test point, and the average value was used to minimize the effect of random fluctuations. Performance metrics like BTE and BSFC were determined using standard thermodynamic calculations, incorporating measured fuel flow, engine output, and the calorific value of the fuel. Emissions, including CO, CO₂, HC, and NOx, were measured using a calibrated exhaust gas analyzer. Instrument error was considered, with the emission readings having a known tolerance of ±2%. To interpret the experimental results, the Taguchi method was applied through Minitab 19 software, using an L_25_ orthogonal array. Signal-to-noise (S/N) ratios were calculated based on the performance objective, either maximizing efficiency or minimizing emissions. The results were analyzed to determine which factors had the most significant influence on engine behavior based on changes in the average S/N ratio across different levels of load, blend, and speed.

### Taguchi optimization technique

The Taguchi method is widely used for optimizing key engine parameters to achieve better efficiency and lower emissions in diesel engines. It applies the principles of Design of Experiments (DOE) to determine the best combination of factors affecting engine performance [26]. A major strength of the Taguchi approach is its ability to deliver reliable results with fewer experimental trials compared to conventional methods. The Taguchi method employs orthogonal arrays and signal-to-noise ratios to simultaneously optimize parameter settings for both performance enhancement and variability reduction. This approach offers a distinct advantage over conventional single-factor testing methods by enabling comprehensive evaluation of multiple interacting variables. By systematically analyzing these interactions, the technique provides deeper insights into complex system behaviors while requiring fewer experimental trials [27]. This approach ensures that the optimized settings remain stable even under changing conditions, leading to more consistent engine behavior. Overall, the method provides a systematic and efficient way to improve process performance and reduce experimental complexity [28]. In this study, the Taguchi optimization method was employed using signal-to-noise (S/N) ratios to determine the most effective parameter settings. Two types of S/N ratios were applied, which are depicted in equations 1 and 2: the “higher-the-better” approach for optimizing brake thermal efficiency (BTE) and CO₂ emissions and the “lower-the-better” method for minimizing brake-specific fuel consumption (BSFC), exhaust gas temperature (EGT), NOx, CO, and HC emissions [29]. Minitab-19 software was used to carry out the analysis. A key component of the Taguchi method is the use of orthogonal arrays, which allow for a systematic investigation of multiple variables, such as fuel blend proportions and load conditions, while minimizing the number of required tests [30]. The selection of 25 experimental runs was guided by the Taguchi L_25_ orthogonal array, which is appropriate for testing three control variables: engine load, biodiesel blend ratio, and speed, each at five levels. This design offers a balanced trade-off between comprehensiveness and experimental feasibility, especially in engine testing, where time, fuel, and wear are limiting factors. Each run was repeated three times, and the results were averaged to improve accuracy and account for variability in measurements. Table 2 presents the experimental matrix detailing the observed performance and emission parameters under varying conditions of engine load, speed, and fuel blend. This structure makes it possible to assess how different parameters influence engine efficiency and emission levels. The technique is especially useful for identifying stable engine settings that maintain performance across varying conditions. By simplifying the experimental design and analyzing interactions between fuel properties and engine behaviour, the Taguchi method offers a practical approach to enhancing engine performance with both diesel and biodiesel blends [31]. In this study, Taguchi analysis was used to examine the effects of three factors engine load, biodiesel blend ratio, and engine speed each tested at five levels. The load levels were set at 20%, 40%, 60%, 80%, and 100%, while the biodiesel blend ratios were 0%, 5%, 10%, 15%, and 20%. Engine speeds were varied from 1300 to 1500 RPM in 50 RPM increments. This experimental setup helps identify the optimal combination of parameters to improve engine performance and reduce emissions efficiently.

**Table 2:** Experimental Matrix with Observed Performance and Emission Parameters under Different Load, Speed, and Blend Conditions.

S.No	Load	Blend	Speed	BTE (%)	BSFC (kg/kWh)	HC (PPM)	CO (%)	NOx (PPM)	CO_2_ (%)
1	20	0	1300	14.467	0.61	27	0.052	389	3.1
2	20	5	1350	14.921	0.59	28	0.054	310	3.4
3	20	10	1400	15.157	0.52	28	0.058	260	4.1
4	20	15	1450	16.032	0.51	30	0.051	436	4.2
5	20	20	1500	16.724	0.5	26	0.049	630	4.3
6	40	0	1350	24.123	0.42	28	0.056	577	4.9
7	40	5	1400	23.722	0.45	32	0.06	460	3.4
8	40	10	1450	24.315	0.43	32	0.066	450	4.4
9	40	15	1500	24.511	0.41	31	0.061	490	4.9
10	40	20	1300	23.921	0.4	30	0.038	614	5.1
11	60	0	1400	28.018	0.39	32	0.04	760	5.5
12	60	5	1450	27.235	0.43	30	0.049	670	4.8
13	60	10	1500	27.891	0.4	33	0.05	665	5.2
14	60	15	1300	28.914	0.31	31	0.038	782	4.7
15	60	20	1350	29.258	0.33	33	0.051	740	5.2
16	80	0	1450	31.354	0.43	31	0.039	930	5.8
17	80	5	1500	30.565	0.37	37	0.057	789	5.3
18	80	10	1300	30.724	0.33	32	0.056	734	6.2
19	80	15	1350	31.012	0.36	33	0.052	810	5.8
20	80	20	1400	31.546	0.4	36	0.047	910	5.3
21	100	0	1500	28.574	0.51	32	0.032	980	7.8
22	100	5	1300	28.916	0.41	34	0.063	756	5.9
23	100	10	1350	29.014	0.42	35	0.067	848	6.2
24	100	15	1400	29.947	0.45	36	0.049	1030	7.4
25	100	20	1450	30.015	0.49	37	0.041	989	6.6

While recent optimization studies increasingly employ Response Surface Methodology (RSM) and machine learning (ML) models for their high predictive capabilities and broad parametric exploration, the Taguchi method remains a highly effective and efficient tool in experimental contexts where the number of trials must be minimized. Given the constraints of physical engine testing, the Taguchi L_25_ orthogonal array offered a balanced and statistically rigorous way to assess three major operating parameters (load, blend, and speed) across five levels each [32]. Moreover, the incorporation of signal-to-noise (S/N) ratios enables robust optimization by accounting for variability and improving result reliability under practical conditions. This method has shown strong precedence in biodiesel-related engine studies and is especially useful when working with limited resources and direct measurements, as was the case in this investigation.

Taguchi S/N ratio for higher is better


$$\begin{array}{c} -10{ln}_{10}\frac{1}{n}\sum_{i=1}^{n}\frac{1}{y_{i}^{2}} \end{array}$$ Eq. 1

Taguchi S/N ratio for lower is better


$$\begin{array}{c} -10{ln}_{10}\frac{1}{n}\sum_{i=1}^{n}y_{i}^{2} \end{array}$$ Eq. 2

The signal-to-noise (S/N) response table outlines how engine performance and emission characteristics are affected by variations in load, fuel blend ratio, and engine speed. For each parameter, such as BTE, BSFC, HC, CO, NOx, and CO₂, Table 3 shows S/N ratios across five levels of the three control factors. BTE was assessed using the “larger-is-better” criterion, while the remaining parameters were evaluated using the “smaller-is-better” approach. Among the three factors, load exhibited the most significant influence across all outputs. It showed the largest variation in S/N ratios for BTE, BSFC, HC, and NOx, indicating that engine load plays a dominant role in determining both efficiency and emissions. The biodiesel blend ratio had a noticeable impact on CO and CO₂ emissions, suggesting that changes in fuel composition affect combustion behavior and the generation of carbon-based pollutants. In contrast, engine speed showed relatively smaller variations in S/N ratios, implying a limited effect on overall performance under the conditions tested. This analysis confirms that the Taguchi method effectively identifies which operating parameters most strongly affect engine performance and emission outcomes, providing valuable guidance for optimizing engine settings when using biodiesel-diesel blends.

**Table 3:** Response Table for Signal-to-Noise Ratios.

Parameter	Level	Load	Blend	Speed
BTE	1	23.77	27.77	27.8
	2	27.65	27.72	27.91
	3	29.02	27.85	27.91
	4	29.84	28.09	28
	5	29.33	28.18	28
	Delta	6.07	0.46	0.2
	Rank	1	2	3
BSFC	1	5.285	6.637	7.96
	2	7.501	7.046	7.631
	3	8.654	7.627	7.138
	4	8.486	7.915	6.807
	5	6.852	7.553	7.242
	Delta	3.369	1.278	1.152
	Rank	1	2	3
HC	1	−28.87	−29.52	−29.75
	2	−29.7	−30.12	−29.9
	3	−30.04	−30.08	−30.28
	4	−30.56	−30.14	−30.08
	5	−30.82	−30.14	−29.99
	Delta	1.95	0.62	0.53
	Rank	1	2	3
CO	1	25.56	27.35	26.31
	2	25.16	24.98	25.08
	3	26.89	24.58	25.98
	4	26.07	26.08	26.32
	5	26.27	26.95	26.26
	Delta	1.73	2.77	1.24
	Rank	2	1	3
NO_x_	1	−51.74	−56.77	−56.06
	2	−54.22	−55.02	−55.83
	3	−57.17	−54.74	−55.72
	4	−58.4	−56.58	−56.33
	5	−59.22	−57.64	−56.8
	Delta	7.48	2.9	1.08
	Rank	1	2	3
CO_2_	1	−11.57	−14.31	−13.74
	2	−13.05	−12.96	−13.97
	3	−14.1	−14.23	−13.91
	4	−15.07	−14.47	−14.12
	5	−16.58	−14.4	−14.62
	Delta	5.01	1.52	0.89
	Rank	1	2	3

### Uncertainty analysis

To ensure the credibility and reproducibility of the results, a comprehensive uncertainty analysis was conducted following the guidelines of the *Guide to the Expression of Uncertainty in Measurement (JCGM 100:2008)* [33]. The combined standard uncertainty (${(U}_{c})$ has been estimated using the Root-Sum-Square (RSS) method, which aggregates independent error sources as equation 1.


$$\begin{array}{c} U_{c}=\;\sqrt{\sum_{i=1}^{n}{\left(u_{i}\right)}^{2}} \end{array}$$ (1)

where $U_{c}$ is the combined standard uncertainty and $u_{i}$ represents the individual uncertainties of measured parameters (e.g., fuel flow rate, dynamometer load, and gas analyzer tolerances). Each test was repeated three times to account for random variability, and average values were used to minimize transient effects. The uncertainty in brake thermal efficiency (BTE) and brake-specific fuel consumption (BSFC) was primarily influenced by fuel flow rate measurement error (±1.0%, manufacturer-specified) and Engine load measurement via eddy current dynamometer (±1.5%). Emission measurements using the AVL gas analyzer (AVL List GmbH) have the tolerances as shown in Table 4.:

**Table 4:** Emission gases and their tolerances.

Gases	Tolerances
Carbon monoxide (CO)	±2.0%
Hydrocarbons (HC)	±2.2%
Nitrogen oxides (NOx)	±2.5%
Carbon dioxide (CO₂)	±2.0%

Table 5 details the step-by-step uncertainty propagation for performance and emission parameters. Individual uncertainties are combined using the RSS method, with total values accounting for repeatability and sensor errors.

**Table 5:** The calculation of overall uncertainties.

Parameter	Measurement Device	Individual Uncertainty${(u}_{i})$	Squared Uncertainty${(u_{i})}^{2}$	Combined Uncertainty${(U}_{c})$	Additional Errors (Repeatability, Sensors)	Total Uncertainty
**BTE**	Fuel flow meter	±1.0%	1.0	1.0 + 2.25 + 0.04=±1.81%1.0 + 2.25 + 0.04=±1.81%	±1.0% (repeatability), ± 0.5% (sensors)	**±2.4%**
	Eddy current dynamometer (load)	±1.5%	2.25			
	LHV of fuel	±0.2%	0.04			
**BSFC**	Fuel flow meter	±1.0%	1.0	1.0 + 2.25=±1.8%1.0 + 2.25=±1.8%	±0.5% (repeatability), ± 0.3% (sensors)	**±2.1%**
	Eddy current dynamometer (load)	±1.5%	2.25			
**CO**	AVL gas analyzer	±2.0%	4.0	4.0=±2.0%4.0=±2.0%	±0.3% (sample conditioning)	**±2.0%**
**HC**	AVL gas analyzer	±2.2%	4.84	4.84=±2.2%4.84=±2.2%	±0.3% (sample conditioning)	**±2.2%**
**NOx**	AVL gas analyzer	±2.5%	6.25	6.25=±2.5%6.25=±2.5%	±0.3% (sample conditioning)	**±2.5%**
**CO₂**	AVL gas analyzer	±2.0%	4.0	4.0=±2.0%4.0=±2.0%	±0.3% (sample conditioning)	**±2.0%**

These estimations align with methodologies found in studies on fuel–nanoparticle interactions and waste-to-fuel combustion analysis. The detailed reporting of uncertainty supports confidence in the optimization results and allows future researchers to replicate similar experiments under controlled conditions [34–36].

## Results and discussions

**Fig 2:**
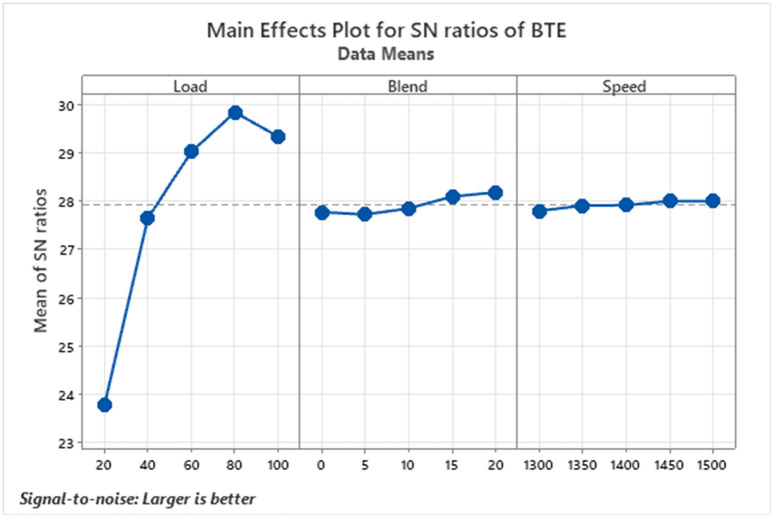
S/N ratios graph for brake thermal efficiency.

Brake Thermal Efficiency (BTE) serves as a key indicator of a diesel engine’s ability to convert the energy in fuel into useful mechanical output. It is calculated by comparing the work produced by the engine to the heat energy supplied by the fuel, shown in Fig 2. A higher BTE value signifies more efficient fuel use and lower energy loss, making it vital for assessing engine performance. In the conducted experiments, BTE improved with increasing biodiesel blend ratios and engine speeds, due to several synergistic factors. Biodiesel contains inherent oxygen within its molecular structure, which facilitates more complete combustion, particularly under high-speed conditions where the available time for combustion is limited.

Additionally, biodiesel generally has a higher cetane number than conventional diesel, resulting in shorter ignition delays and smoother combustion. At higher engine speeds, enhanced air turbulence improves the air-fuel mixing process, which, when combined with biodiesel’s oxygen content, leads to better atomization and more efficient combustion. Among all tested blends, the D80B20 combination consisting of 80% diesel and 20% biodiesel achieved the highest efficiency [37]. BTE also showed a clear upward trend as engine load increased, confirming a positive correlation between load and efficiency. The peak BTE observed was 31.546%, indicating that a 20% biodiesel mix offers optimal efficiency under the test conditions. These observations align with advanced combustion studies indicating that oxygenated fuels enhance thermal efficiency through improved oxidation pathways [14].

**Fig 3:**
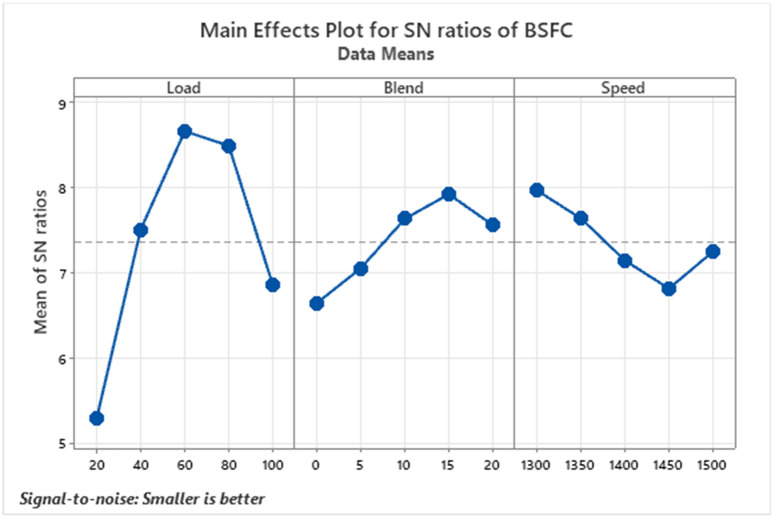
S/N ratios graph for brake-specific fuel consumption.

Brake Specific Fuel Consumption (BSFC) is a fundamental parameter used to assess how efficiently a diesel engine converts fuel into power. It quantifies the amount of fuel required to produce a specific amount of output power, as shown in Fig 3. A lower BSFC indicates that the engine is more efficient in its fuel usage. In the course of testing, BSFC values consistently declined as the biodiesel blend percentage increased [38] due to the improved combustion characteristics of biodiesel. The presence of oxygen in biodiesel molecules enhances the combustion process, allowing for more complete and efficient fuel burning. This increased combustion efficiency means that less fuel is required to produce the same amount of brake power, thereby reducing BSFC. The most noticeable improvement was observed with the D85B15 blend, which recorded the lowest BSFC value of 0.31 g/kWh. Additionally, BSFC showed a downward trend as engine load increased, highlighting an inverse relationship between load and fuel consumption per unit of power. These findings reinforce the link between reduced BSFC and improved Brake Thermal Efficiency (BTE), suggesting that a biodiesel blend, particularly around 15–20%, supports better fuel economy without compromising engine performance.

**Fig 4:**
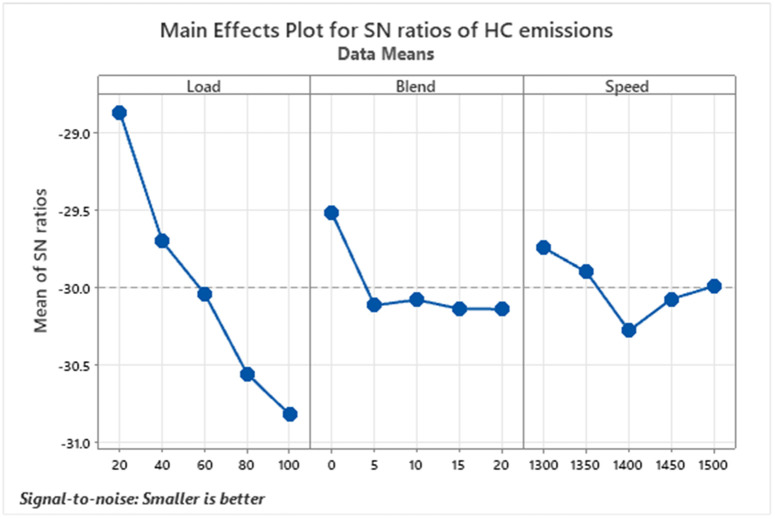
S/N ratios graph for HC emissions.

Hydrocarbon (HC) emissions from diesel engines are a key environmental issue, as they result from unburned or partially burned fuel during the combustion process, as depicted in Fig 4. These emissions contribute to air pollution by forming ground-level ozone and smog, which can aggravate respiratory conditions and pose long-term health risks. Additionally, hydrocarbons play a role in atmospheric chemical reactions, leading to the creation of fine particulate matter and volatile organic compounds, both of which are harmful to human health and contribute to climate change [39]. Experimental results showed that HC emissions tend to increase with higher engine loads due to incomplete combustion under richer air-fuel mixtures. As engine load increases, more fuel is injected to meet the power demand, often leading to locally rich zones within the combustion chamber. These zones may not receive sufficient oxygen for complete combustion, resulting in the formation of unburned hydrocarbons. However, the lowest HC level, 26 ppm, was recorded at 20% load using a D80B20 blend. Overall, a clear decline in hydrocarbon emissions was observed as the proportion of biodiesel in the fuel mix increased, highlighting the effectiveness of biodiesel blends in reducing unburned fuel emissions.

**Fig 5:**
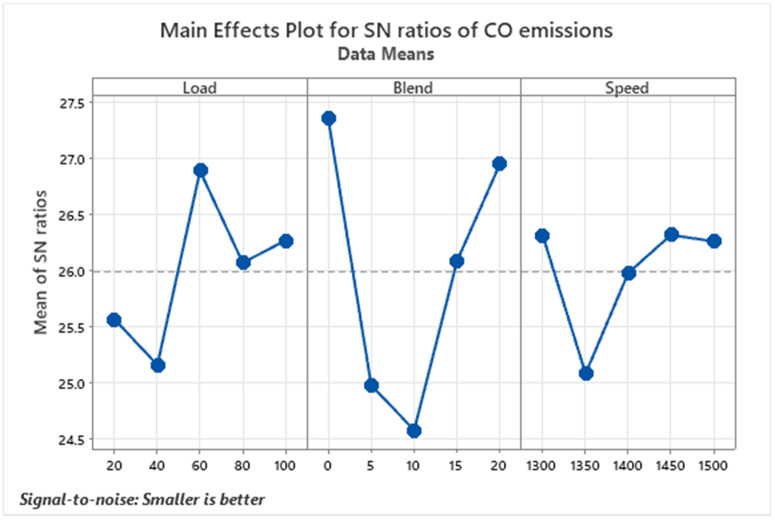
S/N ratios graph for CO emissions.

Carbon Monoxide (CO) Emissions are a major environmental and health concern associated with diesel engine operation. CO forms as a result of incomplete combustion, particularly when there is insufficient oxygen during the fuel-burning process, as illustrated in Fig 5. In the experiments conducted, CO emissions were lowest at pure diesel (D100), with a recorded value of 0.32%. However, a notable rise in CO levels was observed with increased engine loads, peaking at the D90B10 blend under full load conditions [40]. As the engine load increases, more fuel is injected into the combustion chamber to meet the power requirement. However, the available air may not be sufficient to ensure complete oxidation of carbon in the fuel, resulting in the formation of CO. The observed fluctuations in CO emissions under varying engine loads and biodiesel blend ratios result from competing mechanisms governing combustion efficiency and oxidation pathways. At low loads (20–40%), the lean air-fuel mixture provides sufficient oxygen for complete combustion, yielding lower CO emissions. However, the slight dip in CO reduction at 40% load (+25.2 S/N ratio) compared to 20% (+25.5) occurs due to marginally lower cylinder temperatures, which slightly impair combustion efficiency despite excess oxygen availability. The optimal CO reduction at 60% load (+27.0) stems from balanced combustion conditions-adequate temperature, pressure, and residence time—allowing biodiesel’s oxygen content to fully oxidize CO precursors.

At higher loads (80–100%), CO emissions rise again (+26.1 at 80%,  + 26.3 at 100%) due to richer fuel-air mixtures creating localized oxygen-deficient zones. The increased fuel injection shortens combustion residence time, limiting complete CO oxidation. Additionally, peak temperatures promote thermal dissociation of CO₂ back into CO, offsetting biodiesel’s benefits. The non-monotonic CO trend across blend ratios—peaking at 10% biodiesel (S/N  + 24.58) before improving at 15–20% (+26.08 to +26.95)—reflects competing effects of fuel properties. Low biodiesel blends (5–10%) suffer from poor atomization due to higher viscosity, creating fuel-rich pockets that increase CO despite the added oxygen. At higher blends (15–20%), the oxygen content dominates, improving combustion efficiency and CO oxidation. This explains the valley-like trend, which contradicts a simple inverse relationship between biodiesel content and CO emissions.

**Fig 6:**
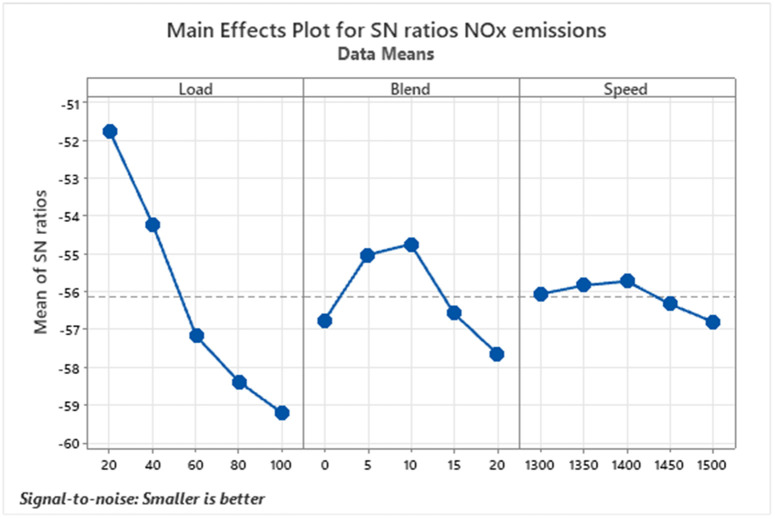
S/N ratios graph for NOx emissions.

Nitrogen oxides (NOx) are harmful pollutants generated during high-temperature combustion in diesel engines, as depicted in Fig 6. These gases, formed from nitrogen and oxygen, are major contributors to air pollution, playing a key role in the development of ground-level ozone, smog, and acid rain. Exposure to NOx can aggravate respiratory illnesses such as asthma, making its reduction a public health priority [41]. Elevated combustion temperatures and specific fuel properties often lead to increased NOx levels. Techniques like Exhaust Gas Recirculation (EGR) are commonly used to mitigate these emissions. In experimental observations, NOx levels dropped to 260 ppm with the D90B10 blend at 20% load while peaking at 1030 ppm under full load using D95B5. Although NOx did not consistently increase with higher biodiesel blend percentages, a clear trend of rising emissions with increasing engine load was evident. However, at lower to moderate blend levels, the increase in NOx may not be significant, as the thermal and chemical effects tend to balance each other out. In some cases, the slightly slower combustion rate and higher viscosity of biodiesel blends can reduce peak temperatures, limiting NOx formation. Additionally, engine load and speed also influence NOx emissions, often having a more dominant impact than the blend ratio alone. As highlighted in foundational reviews, NOx escalation in biodiesel blends stems from adiabatic flame temperature effects, necessitating tailored mitigation like delayed injection or EGR optimization [42].

**Fig 7:**
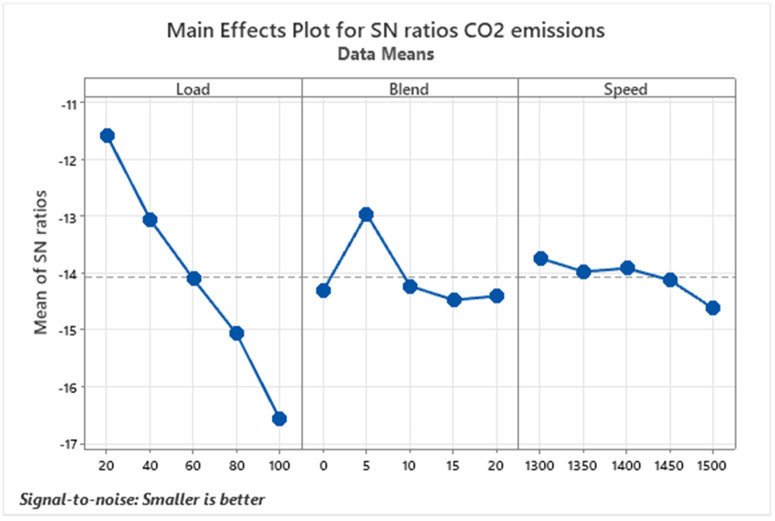
S/N ratios graph for CO_2_ emissions.

Carbon dioxide (CO₂) is a primary greenhouse gas released during the combustion of diesel fuel, contributing significantly to global warming and environmental imbalance, as shown in Fig 7. As diesel burns in an engine, it emits CO₂, a major factor behind the greenhouse effect, which leads to rising global temperatures and disrupted climate patterns [43]. Diesel-powered vehicles and machinery are among the leading sources of CO₂ emissions worldwide, particularly in sectors such as transportation and industry. In experimental observations, CO₂ levels were recorded during full-load operation with pure diesel (D100). Results showed a consistent increase in CO₂ emissions with higher biodiesel blend ratios and load levels, indicating more complete combustion under these conditions. This is due to higher engine loads demanding more fuel, and the increased combustion temperature and pressure under these conditions further support complete combustion processes. The rise in CO₂ emissions under these combined effects reflects the effective utilization of the fuel’s energy content, even though it also suggests increased carbon output as a trade-off for better combustion efficiency.

To facilitate a comparative assessment of emissions relative to engine output and fuel energy content, Table 6 summarizes the normalized emission results in kg/kWh for all tested conditions. This normalization accounts for variations in engine load and fuel blend ratios, providing a clearer evaluation of real-time performance. Key trends include: (1) NOx emissions increase with higher loads but exhibit sensitivity to biodiesel content; (2) CO and HC reductions correlate with higher biodiesel blends due to oxygen-enhanced combustion; and (3) CO₂ levels remain stable across blends but rise with load, reflecting complete oxidation. These findings complement the earlier ppm/% data while offering actionable insights for practical engine applications.

**Table 6:** Normalized Emission Results (kg/kWh) for Different Engine Loads, Blends, and Speeds.

S. No	Load (%)	Blend (D/B)	Speed (RPM)	HC (kg/kWh)	CO (kg/kWh)	NOx (kg/kWh)	CO₂ (kg/kWh)
1	20	D100 (0% B)	1300	0.0012	0.0023	0.0171	0.1364
2	20	D95B5 (5% B)	1350	0.0013	0.0025	0.0143	0.1568
3	20	D90B10 (10% B)	1400	0.0013	0.0027	0.0120	0.1892
4	20	D85B15 (15% B)	1450	0.0014	0.0024	0.0201	0.1936
5	20	D80B20 (20% B)	1500	0.0012	0.0023	0.0290	0.1982
6	40	D100 (0% B)	1350	0.0013	0.0026	0.0266	0.2258
7	40	D95B5 (5% B)	1400	0.0015	0.0028	0.0212	0.1568
8	40	D90B10 (10% B)	1450	0.0015	0.0030	0.0207	0.2028
9	40	D85B15 (15% B)	1500	0.0014	0.0028	0.0226	0.2258
10	40	D80B20 (20% B)	1300	0.0014	0.0018	0.0283	0.2350
11	60	D100 (0% B)	1400	0.0014	0.0018	0.0350	0.2535
12	60	D95B5 (5% B)	1450	0.0014	0.0023	0.0309	0.2212
13	60	D90B10 (10% B)	1500	0.0015	0.0023	0.0306	0.2396
14	60	D85B15 (15% B)	1300	0.0014	0.0018	0.0360	0.2166
15	60	D80B20 (20% B)	1350	0.0015	0.0023	0.0341	0.2396
16	80	D100 (0% B)	1450	0.0014	0.0018	0.0428	0.2673
17	80	D95B5 (5% B)	1500	0.0017	0.0026	0.0364	0.2442
18	80	D90B10 (10% B)	1300	0.0015	0.0026	0.0338	0.2857
19	80	D85B15 (15% B)	1350	0.0015	0.0024	0.0373	0.2673
20	80	D80B20 (20% B)	1400	0.0017	0.0022	0.0419	0.2442
21	100	D100 (0% B)	1500	0.0015	0.0015	0.0452	0.3595
22	100	D95B5 (5% B)	1300	0.0016	0.0029	0.0348	0.2719
23	100	D90B10 (10% B)	1350	0.0016	0.0031	0.0391	0.2857
24	100	D85B15 (15% B)	1400	0.0017	0.0023	0.0475	0.3410
25	100	D80B20 (20% B)	1450	0.0017	0.0019	0.0456	0.3042

## Conclusions

This study explored the performance and emission characteristics of a single-cylinder diesel engine using blends of mahua biodiesel and conventional diesel fuel. Engine load, blend ratio, and speed were varied systematically to assess their individual and combined effects on key metrics such as BTE, BSFC, and exhaust emissions. Among the tested configurations, the D80B20 blend delivered the highest thermal efficiency, while D85B15 showed the lowest BSFC, indicating effective fuel utilization. Emission analysis revealed that increasing biodiesel content generally contributed to lower CO and HC emissions, while NOx and CO_2_ showed an upward trend, particularly at higher loads. While the results support the suitability of mahua biodiesel as a supplementary fuel, several practical challenges were noted. Variability in combustion at different speeds and the impact of biodiesel viscosity on injection and atomization were observed during testing. To build on these findings, future work should investigate long-term engine performance using biodiesel blends, focusing on durability, maintenance cycles, and cold-start behavior. Given the use of an underutilized non-edible feedstock, a multi-response optimization approach, and commercially sourced biodiesel without further refinement, this study offers practical insights into the real-world integration of biodiesel in existing diesel engines. Further work should also explore the scalability and lifecycle sustainability of mahua biodiesel for widespread deployment. Additional research is also needed to evaluate the scalability of mahua biodiesel production and its economic viability in large-scale applications. Future assessments should adopt integrated frameworks evaluating socioeconomic viability and agroecological impacts alongside emissions. A full life cycle assessment, incorporating environmental, economic, and energy return metrics, would provide a more comprehensive understanding of its sustainability.

## Supporting information

S1 Data Data Availability. (DOCX)
