# Supplementary material for: Optimizing diesel engine performance and emissions with mahua biodiesel blends using taguchi methodology
Source: PLoS One. 2025 Sep 5;20(9):e0332035. doi: 10.1371/journal.pone.0332035 (PMC12412990; doi:10.1371/journal.pone.0332035)
Supplement: S1 Data — (DOCX) [file pone.0332035.s001.docx]

Table 1: Engine specifications

| Number of Cylinders | 1 |
| --- | --- |
| Number of Strokes | 4 |
| Stroke Length | 110 mm |
| Connecting rod length | 234 mm |
| Orifice Diameter | 20 mm |
| Dynamometer length | 185 mm |
| Fuel | Diesel |
| Power | 3.5 kW |
| Compression Ratio | 18 |
| Dynamometer Type | Eddy Current |

Table 2: Experimental Matrix with Observed Performance and Emission Parameters under Different Load, Speed, and Blend Conditions

| **S.No** | **Load** | **Blend** | **Speed** | **BTE (%)** | **BSFC (kg/kwh)** | **HC (PPM)** | **CO**  **(%)** | **NOx**  **(PPM)** | **CO_2_ (%)** |
| --- | --- | --- | --- | --- | --- | --- | --- | --- | --- |
| 1 | 20 | 0 | 1300 | 14.467 | 0.61 | 27 | 0.052 | 389 | 3.1 |
| 2 | 20 | 5 | 1350 | 14.921 | 0.59 | 28 | 0.054 | 310 | 3.4 |
| 3 | 20 | 10 | 1400 | 15.157 | 0.52 | 28 | 0.058 | 260 | 4.1 |
| 4 | 20 | 15 | 1450 | 16.032 | 0.51 | 30 | 0.051 | 436 | 4.2 |
| 5 | 20 | 20 | 1500 | 16.724 | 0.5 | 26 | 0.049 | 630 | 4.3 |
| 6 | 40 | 0 | 1350 | 24.123 | 0.42 | 28 | 0.056 | 577 | 4.9 |
| 7 | 40 | 5 | 1400 | 23.722 | 0.45 | 32 | 0.06 | 460 | 3.4 |
| 8 | 40 | 10 | 1450 | 24.315 | 0.43 | 32 | 0.066 | 450 | 4.4 |
| 9 | 40 | 15 | 1500 | 24.511 | 0.41 | 31 | 0.061 | 490 | 4.9 |
| 10 | 40 | 20 | 1300 | 23.921 | 0.4 | 30 | 0.038 | 614 | 5.1 |
| 11 | 60 | 0 | 1400 | 28.018 | 0.39 | 32 | 0.04 | 760 | 5.5 |
| 12 | 60 | 5 | 1450 | 27.235 | 0.43 | 30 | 0.049 | 670 | 4.8 |
| 13 | 60 | 10 | 1500 | 27.891 | 0.4 | 33 | 0.05 | 665 | 5.2 |
| 14 | 60 | 15 | 1300 | 28.914 | 0.31 | 31 | 0.038 | 782 | 4.7 |
| 15 | 60 | 20 | 1350 | 29.258 | 0.33 | 33 | 0.051 | 740 | 5.2 |
| 16 | 80 | 0 | 1450 | 31.354 | 0.43 | 31 | 0.039 | 930 | 5.8 |
| 17 | 80 | 5 | 1500 | 30.565 | 0.37 | 37 | 0.057 | 789 | 5.3 |
| 18 | 80 | 10 | 1300 | 30.724 | 0.33 | 32 | 0.056 | 734 | 6.2 |
| 19 | 80 | 15 | 1350 | 31.012 | 0.36 | 33 | 0.052 | 810 | 5.8 |
| 20 | 80 | 20 | 1400 | 31.546 | 0.4 | 36 | 0.047 | 910 | 5.3 |
| 21 | 100 | 0 | 1500 | 28.574 | 0.51 | 32 | 0.032 | 980 | 7.8 |
| 22 | 100 | 5 | 1300 | 28.916 | 0.41 | 34 | 0.063 | 756 | 5.9 |
| 23 | 100 | 10 | 1350 | 29.014 | 0.42 | 35 | 0.067 | 848 | 6.2 |
| 24 | 100 | 15 | 1400 | 29.947 | 0.45 | 36 | 0.049 | 1030 | 7.4 |
| 25 | 100 | 20 | 1450 | 30.015 | 0.49 | 37 | 0.041 | 989 | 6.6 |

Table 3: Response Table for Signal to Noise Ratios

| **Parameter** | **Level** | **Load** | **Blend** | **Speed** |
| --- | --- | --- | --- | --- |
| BTE | 1 | 23.77 | 27.77 | 27.8 |
|  | 2 | 27.65 | 27.72 | 27.91 |
|  | 3 | 29.02 | 27.85 | 27.91 |
|  | 4 | 29.84 | 28.09 | 28 |
|  | 5 | 29.33 | 28.18 | 28 |
|  | Delta | 6.07 | 0.46 | 0.2 |
|  | Rank | 1 | 2 | 3 |
| BSFC | 1 | 5.285 | 6.637 | 7.96 |
|  | 2 | 7.501 | 7.046 | 7.631 |
|  | 3 | 8.654 | 7.627 | 7.138 |
|  | 4 | 8.486 | 7.915 | 6.807 |
|  | 5 | 6.852 | 7.553 | 7.242 |
|  | Delta | 3.369 | 1.278 | 1.152 |
|  | Rank | 1 | 2 | 3 |
| HC | 1 | -28.87 | -29.52 | -29.75 |
|  | 2 | -29.7 | -30.12 | -29.9 |
|  | 3 | -30.04 | -30.08 | -30.28 |
|  | 4 | -30.56 | -30.14 | -30.08 |
|  | 5 | -30.82 | -30.14 | -29.99 |
|  | Delta | 1.95 | 0.62 | 0.53 |
|  | Rank | 1 | 2 | 3 |
| CO | 1 | 25.56 | 27.35 | 26.31 |
|  | 2 | 25.16 | 24.98 | 25.08 |
|  | 3 | 26.89 | 24.58 | 25.98 |
|  | 4 | 26.07 | 26.08 | 26.32 |
|  | 5 | 26.27 | 26.95 | 26.26 |
|  | Delta | 1.73 | 2.77 | 1.24 |
|  | Rank | 2 | 1 | 3 |
| NO_x_ | 1 | -51.74 | -56.77 | -56.06 |
|  | 2 | -54.22 | -55.02 | -55.83 |
|  | 3 | -57.17 | -54.74 | -55.72 |
|  | 4 | -58.4 | -56.58 | -56.33 |
|  | 5 | -59.22 | -57.64 | -56.8 |
|  | Delta | 7.48 | 2.9 | 1.08 |
|  | Rank | 1 | 2 | 3 |
| CO_2_ | 1 | -11.57 | -14.31 | -13.74 |
|  | 2 | -13.05 | -12.96 | -13.97 |
|  | 3 | -14.1 | -14.23 | -13.91 |
|  | 4 | -15.07 | -14.47 | -14.12 |
|  | 5 | -16.58 | -14.4 | -14.62 |
|  | Delta | 5.01 | 1.52 | 0.89 |
|  | Rank | 1 | 2 | 3 |

### **Table 4: Normalized Emission Results (kg/kWh) for Different Engine Loads, Blends, and Speeds**

| **S. No** | **Load (%)** | **Blend (D/B)** | **Speed (RPM)** | **HC (kg/kWh)** | **CO (kg/kWh)** | **NOx (kg/kWh)** | **CO₂ (kg/kWh)** |
| --- | --- | --- | --- | --- | --- | --- | --- |
| 1 | 20 | D100 (0% B) | 1300 | 0.0012 | 0.0023 | 0.0171 | 0.1364 |
| 2 | 20 | D95B5 (5% B) | 1350 | 0.0013 | 0.0025 | 0.0143 | 0.1568 |
| 3 | 20 | D90B10 (10% B) | 1400 | 0.0013 | 0.0027 | 0.0120 | 0.1892 |
| 4 | 20 | D85B15 (15% B) | 1450 | 0.0014 | 0.0024 | 0.0201 | 0.1936 |
| 5 | 20 | D80B20 (20% B) | 1500 | 0.0012 | 0.0023 | 0.0290 | 0.1982 |
| 6 | 40 | D100 (0% B) | 1350 | 0.0013 | 0.0026 | 0.0266 | 0.2258 |
| 7 | 40 | D95B5 (5% B) | 1400 | 0.0015 | 0.0028 | 0.0212 | 0.1568 |
| 8 | 40 | D90B10 (10% B) | 1450 | 0.0015 | 0.0030 | 0.0207 | 0.2028 |
| 9 | 40 | D85B15 (15% B) | 1500 | 0.0014 | 0.0028 | 0.0226 | 0.2258 |
| 10 | 40 | D80B20 (20% B) | 1300 | 0.0014 | 0.0018 | 0.0283 | 0.2350 |
| 11 | 60 | D100 (0% B) | 1400 | 0.0014 | 0.0018 | 0.0350 | 0.2535 |
| 12 | 60 | D95B5 (5% B) | 1450 | 0.0014 | 0.0023 | 0.0309 | 0.2212 |
| 13 | 60 | D90B10 (10% B) | 1500 | 0.0015 | 0.0023 | 0.0306 | 0.2396 |
| 14 | 60 | D85B15 (15% B) | 1300 | 0.0014 | 0.0018 | 0.0360 | 0.2166 |
| 15 | 60 | D80B20 (20% B) | 1350 | 0.0015 | 0.0023 | 0.0341 | 0.2396 |
| 16 | 80 | D100 (0% B) | 1450 | 0.0014 | 0.0018 | 0.0428 | 0.2673 |
| 17 | 80 | D95B5 (5% B) | 1500 | 0.0017 | 0.0026 | 0.0364 | 0.2442 |
| 18 | 80 | D90B10 (10% B) | 1300 | 0.0015 | 0.0026 | 0.0338 | 0.2857 |
| 19 | 80 | D85B15 (15% B) | 1350 | 0.0015 | 0.0024 | 0.0373 | 0.2673 |
| 20 | 80 | D80B20 (20% B) | 1400 | 0.0017 | 0.0022 | 0.0419 | 0.2442 |
| 21 | 100 | D100 (0% B) | 1500 | 0.0015 | 0.0015 | 0.0452 | 0.3595 |
| 22 | 100 | D95B5 (5% B) | 1300 | 0.0016 | 0.0029 | 0.0348 | 0.2719 |
| 23 | 100 | D90B10 (10% B) | 1350 | 0.0016 | 0.0031 | 0.0391 | 0.2857 |
| 24 | 100 | D85B15 (15% B) | 1400 | 0.0017 | 0.0023 | 0.0475 | 0.3410 |
| 25 | 100 | D80B20 (20% B) | 1450 | 0.0017 | 0.0019 | 0.0456 | 0.3042 |
